# First record of the aphid genus *Jacksonia* Theobald (Hemiptera, Aphididae, Aphidinae) from China, with description of one new species

**DOI:** 10.3897/zookeys.312.5506

**Published:** 2013-06-26

**Authors:** Xing-Yi Li, Bin Zhang, Xiao-Mei Su, Ge-Xia Qiao

**Affiliations:** 1 Key Laboratory of Zoological Systematics and Evolution, Institute of Zoology, Chinese Academy of Sciences, No. 1 Beichen West Road, Chaoyang District, Beijing 100101, P.R.China; 2 University of Chinese Academy of Sciences, No. 19 Yuquan Road, Shijingshan District, Beijing 100049, P.R.China

**Keywords:** *Jacksonia*, Aphididae, new record genus, new species, China

## Abstract

The aphid genus *Jacksonia* Theobald is reported in China for the first time, from Shaanxi, with the description of a new species, *Jacksonia gibbera* Qiao, Li, Zhang & Su, **sp. n.**, found on an unidentified plant belonging to the Leguminosae/Fabaceae. A key to species of *Jacksonia* is provided.

## Introduction

The aphid genus *Jacksonia* was erected by Theobald (1923), with a description of the type species *Jacksonia papillata*. It was distinguished by the peculiar shape of the siphunculi, constricted at the middle, without a flange or any reticulation, and well-developed antennal tubercles which are converging, rough and very broad. Until now, this genus is represented by only three known species in the world (Ghosh 1986; Heie 1994; Raychaudhuri 1980; Remaudière and Remaudière 1997; Favret 2013). After identifying the specimens from Shaanxi, China, and comparing the original descriptions of the known species, one new species of *Jacksonia* is reported from China, *Jacksonia gibbera* Qiao, Li, Zhang & Su, sp. n. It was collected on an unidentified species of Leguminosae (Fabaceae).

## Taxonomy

### Jacksonia

Theobald, 1923

http://species-id.net/wiki/Jacksonia

Jacksonia Theobald 1923: 19. Type species: *Jacksonia papillata* Theobald, 1923; 19–20 & Figs A–E, by monotypy.
Jacksonia Theobald: Eastop and Hille Ris Lambers 1976: 229; Miyazaki 1971: 112; Raychaudhuri 1980: 154; Ghosh 1986: 92; Heie 1994: 43; Remaudière and Remaudière 1997: 105; Blackman and Eastop 2006: 1183.

#### Generic diagnosis.

In apterous viviparous female: Body broadly elongate. Head scabrous, with dense spinules or warts on dorsum and venter. Antennal tubercles well developed, strongly converging, broad and covered with many warts; medial frontal tubercle indistinct. Antennae 6-segmented, shorter than body, antennal segments I-V with distinct warty imbrications, primary rhinaria non-ciliated or ciliated. Ultimate rostral segment wedge-shaped, with 2 or 3 accessory setae. Distal 2/3 of femora and bases of tibiae with warty imbrications, hind tibiae of nymphs without spinules. First tarsal chaetotaxy 3, 3, 2, or 3, 3, 3. Antennal and dorsal body setae short, blunt or acute at apex. Dorsum of body scabrous; pale or with dark bands on abdominal tergites VI-VII. Siphunculi cylindrical, wide at base, narrow at middle and again becoming wide, with oblique or central aperture lacking a flange, with scabrous imbrications. Distance between 6^th^ and 7^th^ spiracles much less than that between 5^th^ and 6^th^. Cauda tongue-shaped, with blunt apex, shorter than siphunculi, with 4-6 setae. Anal and genital plates broadly circular, genital plate with 6-19 posterior setae and 2 anterior setae.

In alate viviparous female: Dorsum of head smooth or sparsely to densely spinulose, venter smooth. Frontal setae with acute apices. Antennal segments I-V with normal imbrications, segments III-V with small or large round or transverse oval secondary rhinaria. Fore wings with two-forked medial veins, hind wings with 2 oblique veins. Abdominal tergites with brown or blackish brown spino-pleural and marginal patches, spino-pleural patches on tergites III-V usually fused to form a large dorsal patch. Others similar to apterae.

#### Distribution.

India, Japan, Europe and newly recorded from China (Shaanxi). In Blackman and Eastop (aphidsonworldsplants.info): India (*Jacksonia campanulata*); in regions with temperate oceanic climates throughout the world, including many oceanic islands (e.g. Iceland, Faroes, Azores, Auckland Is., Macquarie Is., South Georgia) (*Jacksonia papillata*).

#### Host plants.

On various species of Poaceae (*Dactylis*, *Deschampsia*, *Festuca*, *Poa*), but also with species described from *Campanula* and an unidentified plant belonging to the Leguminosae/Fabaceae. Other recorded hosts are likely to be casual occurrences (Blackman and Eastop 2006).

#### Comments.

This genus is related to *Myzus* Passerini, 1860, but apterae can be distinguished from the latter by the peculiarly shaped siphunculi and the very broad antennal tubercles. The genus is also very similar to *Xenosiphonaphis* Takahashi, 1961, in having very broad antennal tubercles and flangeless siphunculi, but in *Xenosiphonaphis*, the inner apex of the antennal tubercles is only slightly converging. The alatae also differ: in *Jacksonia*, the basal halves of the siphunculi are without spinules, transverse wrinkles or grooves while those of *Xenosiphonaphis* have transverse wrinkles or grooves on the basal half.

### Jacksonia gibbera

Qiao, Li, Zhang & Su sp. n.

urn:lsid:zoobank.org:act:50411893-02D1-4A72-80BB-C97308737D08

http://species-id.net/wiki/Jacksonia_gibbera

Figures 1 – 27

#### Locus typicus.

China (Shaanxi, altitude 3620 m).

#### Etymology.

The new species is named for the slightly elongated distal half of the siphunculi; “*gibbera*” (Latin) means “gibbous or humpy”.

#### Specimens examined.

Holotype: apterous viviparous female, **CHINA:** Shaanxi (Zhouzhi County, Houzhenzi Town, Mount Qinling, altitude 3620 m), 21 Jun. 1999, No. 12309-1-1, on an unidentified plantof Leguminosae (Fabaceae), coll. T.L. He. *Paratypes*: 2 apterous viviparous females and 4 nymphs, with the same collection data as holotype.

#### Description.

*Apterous viviparous females*: Body oval, 1.42–1.62 mm long, 0.82–0.95 mm wide. Green in life. In mounted specimens, body pale; apical parts of base of antennal segment VI and processus terminalis, apex of rostrum and tarsi brown, other parts pale (Fig. 13).

**Figures 1–12.**
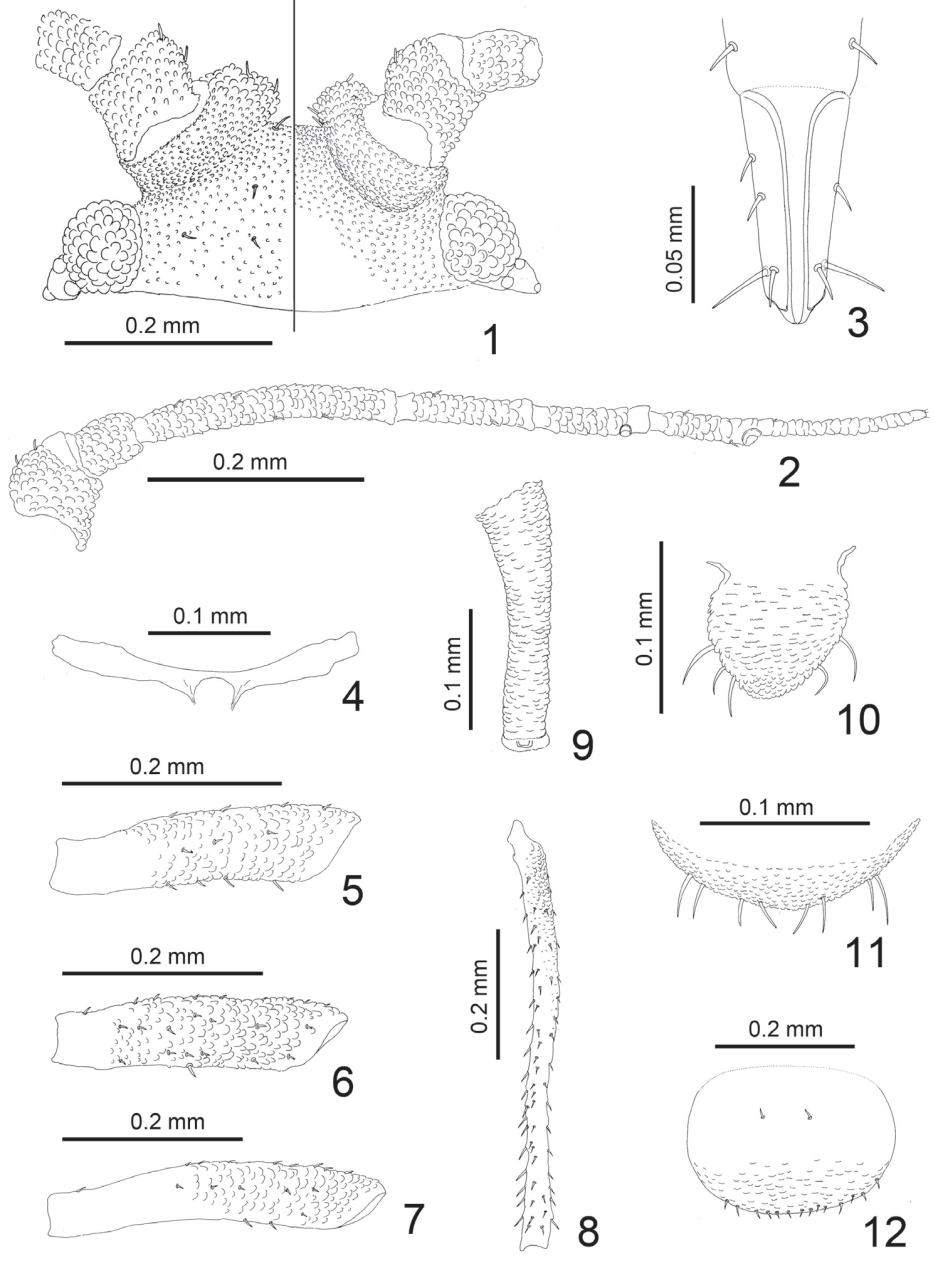
*Jacksonia gibbera* Qiao, Li, Zhang & Su, sp. n. Apterous viviparous female: **1** dorsal (left) and ventral (right) views of head **2** antenna **3** ultimate rostral segment **4** mesosternal furca **5** fore femur **6** mid-femur **7** hind femur **8** hind tibia **9** siphunculus **10** cauda **11** anal plate **12** genital plate.

*Head*: Head scabrous, with dense spinules or warts on dorsum and venter, but warts on dorsal median area of head are sparse (Figs 1, 14). Dorsal setae of head very short, blunt or slightly expanded at apex. Head with one pair of frontal setae and two pairs on antennal tubercles, two pairs of dorsal setae between antennae, two pairs of dorsal setae between eyes. Frontal setae approximately as long as dorsal setae of head, 0.005–0.012 mm, 0.2–0.5 of basal diameter of antennal segment III. Antennal tubercles well developed, very broad, converging, with dense warts; medial frontal tubercle indistinct (Figs 1, 14). Antennae 6-segmented, segments I-V and base of segment VI with distinct warty imbrications (Figs 2, 15), processus terminalis with weak imbrications (Fig. 16), segment III slightly constricted at base (Figs 2, 15); antenna 0.87–0.88 mm long, 0.54–0.61 times length of body; segment III 0.26–0.27 mm, lengths of segments I-VI: 27-31, 19-22, 100, 46-47, 42-46, 32-38+52-61, respectively; processus terminalis 1.60–1.77 times base of segment. Antennal setae short and blunt, segments I-VI each with 3, 1, 6-7, 4, 4, 2+3 setae, respectively, apex of processus terminalis with three short setae; length of setae on segment III 0.005–0.007 mm, 0.22–0.30 times as long as basal diameter of the segment. Primary rhinaria not ciliated, secondary rhinaria absent (Fig. 2). Rostrum (Figs 3, 18) reaching between mid- and hind coxae, ultimate rostral segment wedge-shaped, 0.11–0.12 mm long, 2.15–2.53 times as long as its basal width, 1.30–1.44 times as long as second hind tarsal segment, with three pairs of primary setae and two or three accessory setae.

*Thorax*: Dorsum of thorax with wrinkles, more distinct toward marginal area of body; pronotum with one pair of spinal and one pair pleural setae, respectively, marginal setae indistinct; metanotum with one pair of spinal, two pairs of pleural and one pair of marginal setae, respectively. Mesosternal furca with a short stem (Figs 4, 17). Spiracles small, reniform-shaped, closed; distance between 6^th^ and 7^th^ spiracles much less than that between 5^th^ and 6^th^. Legs: distal 2/3–4/5 part of femora with distinct warty imbrications (Figs 5–7, 21–23), outer of basal part of tibia with warts (Figs 8, 24), others smooth. Hind femur 0.35–0.38 mm long, 1.28–1.46 times antennal segment III; hind tibia 0.55–0.61 mm long, 0.35–0.42 times length of body; setae on hind tibia long, thick and pointed, 0.025–0.030 mm long, 0.77–1.00 times as long as middle width of the segment. First tarsal chaetotaxy: 3, 3, 3. Second hind tarsal segment 0.08 mm long.

**Figures 13–20.**
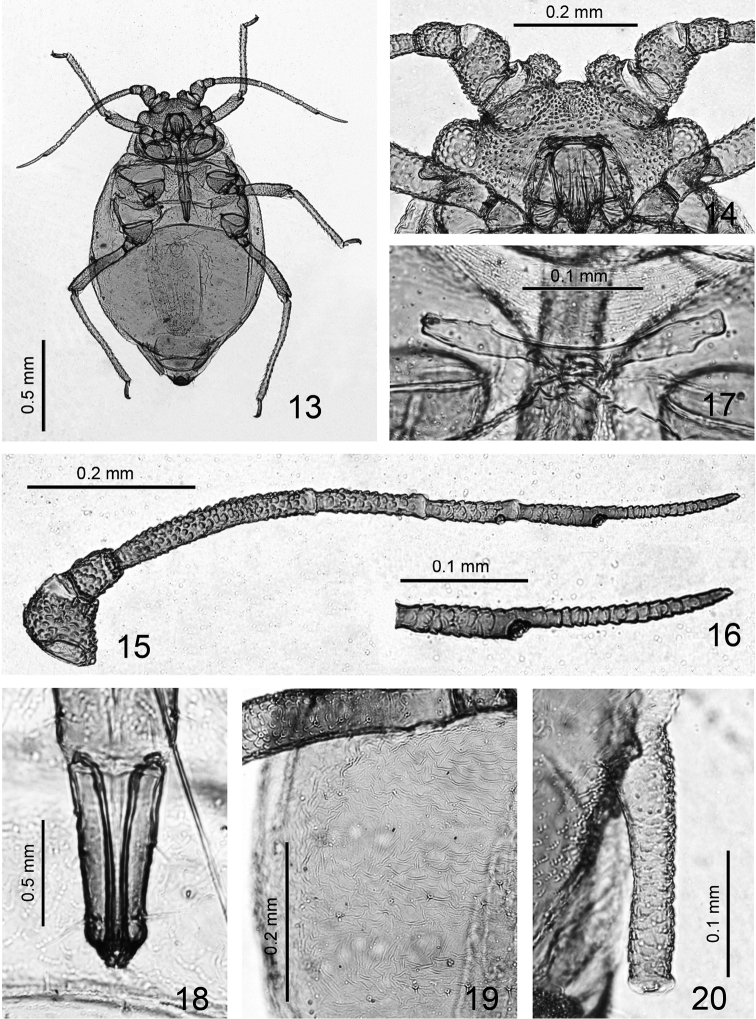
*Jacksonia gibbera* Qiao, Li, Zhang & Su, sp. n. Apterous viviparous female: **13** whole body **14** ventral view of head, antennal tubercles and antennal segments I-II **15** antenna **16** antennal segment VI **17** mesosternal furca **18** ultimate rostral segment **19** detail of abdominal cuticle **20** siphunculus.

*Abdomen*: Abdominal tergites I-VI with wrinkles, more distinct toward marginal area of body (Fig. 19); posterior area of siphunculi with distinct spinules, tergites VII-VIII with sparsely spinulose transverse stripes; venter with spinulose transverse stripes. Dorsal setae of body very short, blunt or slightly capitate at apex, ventral setae short and acute. Abdominal tergites I-VII each with one pair of spinal setae, tergites I, V-VII each with one pair of marginal setae, tergites II-III each with two pairs of marginal setae, tergite IV with three pairs of marginal setae; tergite VIII with two dorsal setae. Length of marginal setae on tergite I about as long as dorsal setae on tergite VIII, 0.005 mm, 0.2 of basal diameter of antennal segment III. Siphunculi cylindrical (Figs 9, 20), constricted in middle, inner side of distal half slightly elongated; with dense imbrications, flangeless, ends of siphunculi truncated, with pore in central area; 0.20–0.21 mm long, 0.13–0.14 times as long as body, 3.42–4.32 times as long as its basal width, 2.2–2.3 times cauda. Cauda tongue-shaped, blunt at apex (Figs 10, 25), 0.09–0.094 mm long, 0.075–0.088 mm wide, with 4–5 setae. Anal plate semi-circular (Fig. 11), with 10 setae. Genital plate broadly circular (Figs 12, 26), with 10–14 posterior setae and two anterior setae.

Hind tibiae in immatures (third instar) without spinules, smooth (Fig. 27).

**Figures 21–27.**
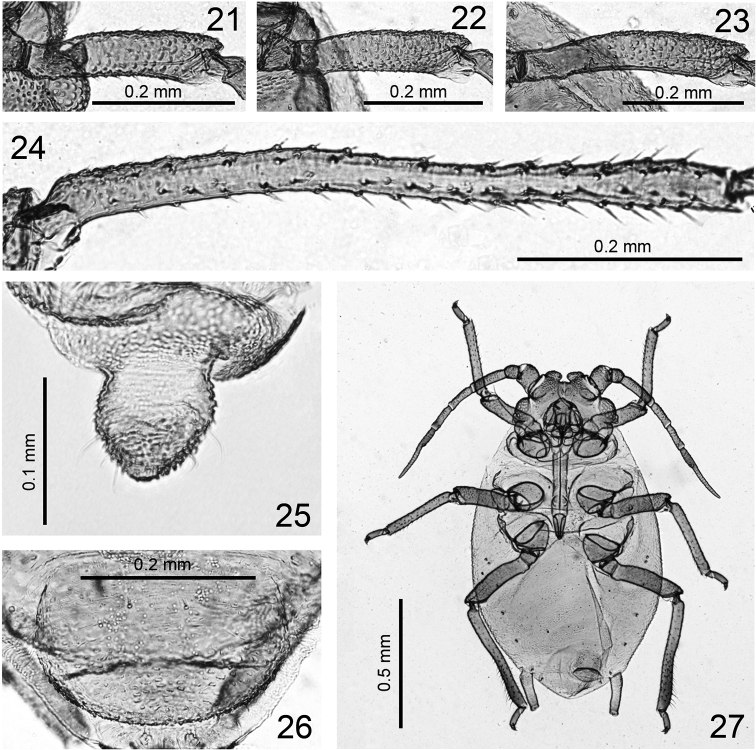
*Jacksonia gibbera* Qiao, Li, Zhang & Su, sp. n. Apterous viviparous female: **21** hind femur **22** mid-femur **23** hind femur **24** hind tibia **25** cauda **26** genital plate **27** 3th instar nymph.

#### Host plant.

An unidentified plantbelonging to the Leguminosae/Fabaceae. It colonises the undersides of the leaves of the host plant.

#### Taxonomic notes.

The new species is similar to the type species *Jacksonia papillata* Theobald, but apterae differ from this and *Jacksonia campanulata* by the characters given in the key below. The fourth species, *Jacksonia sikkimensis* Ghosh, Basu & Raychaudhuri, is known only from alate viviparous females.

##### Key to species of *Jacksonia* (apterous viviparous females)

<br/>

**Table:** 

1	Antennae shorter than 0.6 times body length; siphunculi constricted in middle, with distinct sculpture or spinules; abdominal tergites with wrinkles, polygonal reticulations absent	2
–	Antennae approximately 0.8 times body length; siphunculi subcylindrical without any medial constriction, smooth without sculptures or spinules; abdominal tergites with distinct polygonal reticulations (based on Chakrabarti and Raychaudhuri 1978: 97)	*Jacksonia campanulata* Chakrabarti & Raychaudhuri
2	Siphunculi 1.5–1.8 times cauda, scaly, with a rather small terminal, slightly oblique aperture (turned inwards); dorsum of head with dense warts uniformly distributed; primary rhinaria ciliated; only intersegmental abdominal areas with wrinkles; first tarsal chaetotaxy 3, 3, 2 (based on Ghosh 1986: 92–93; Heie 1994: 43–44)	*Jacksonia papillata* Theobald
–	Siphunculi about 2.2-2.3 times cauda, with imbrications, slightly expanded on inner side of distal half, with a terminal, central aperture; dorsal median area of head with few warts; primary rhinaria not ciliated; whole abdominal tergites with wrinkles; first tarsal chaetotaxy 3, 3, 3	*Jacksonia gibbera* Qiao, Li, Zhang & Su, sp.n.

## Supplementary Material

XML Treatment for Jacksonia

XML Treatment for Jacksonia gibbera
